# Cytotoxic Effects of Ochratoxin A in Neuro-2a Cells: Role of Oxidative Stress Evidenced by *N*-acetylcysteine

**DOI:** 10.3389/fmicb.2016.01142

**Published:** 2016-08-02

**Authors:** Pratiksha V. Bhat, Pandareesh, Farhath Khanum, Anand Tamatam

**Affiliations:** Biochemistry and Nano Sciences Division, Defence Food Research LaboratoryMysore, India

**Keywords:** Ochratoxin-A, Neuro-2a, *N*-acetylcysteine, reactive oxygen species, c-Jun N-terminal kinases, apoptosis

## Abstract

Ochratoxin-A (OTA), is toxic secondary metabolite and is found to be a source of vast range of toxic effects like hepatotoxicity, nephrotoxicity. However, the information available currently regarding neurotoxic effects exerted by OTA is scanty. Hence, the present study was aimed to evaluate the neurotoxic effects of OTA and the possible mechanisms of toxicity as well as the role of cytotoxic oxidative stress on neuronal (Neuro-2a) cell line was evaluated *in vitro*. Results of the MTT and LDH assay showed that, OTA induced dose-dependent cell death in Neuro-2a cells and EC50 value was determined as 500 nM. OTA induced high levels of reactive oxygen species (ROS) and elevated levels of malondialdehyde, also loss of mitochondrial membrane potential was observed in a dose depended manner. Effects of OTA on ROS induced chromosomal DNA damage was assessed by Comet assay and plasmid DNA damage assay in which increase in DNA damage was observed in Neuro-2a cells by increasing the OTA concentration. Further western blotting analysis of OTA treated Neuro-2a cells indicated elevated expression levels of c-Jun, JNK3 and cleaved caspase-3 leading to apoptotic cell death. Other hand realtime-Q-PCR analysis clearly indicates the suppressed expression of neuronal biomarker genes including AChE, BDNF, TH and NOS2. Further *N*-acetylcysteine (NAC) pretreatment to Neuro-2a cells followed by OTA treatment clearly evidenced that, the significant reversal of toxic effects exerted by OTA on Neuro-2a cells. In the present study, results illustrate that ROS a principle event in oxidative stress was elevated by OTA toxicity in Neuro-2a cells. However, further *in vivo*, animal studies are in need to conclude the present study reports and the use of NAC as a remedy for OTA induced neuronal stress.

## Introduction

Ochratoxin, is produced by filamentous fungi species *Aspergillus* and *Penicillium* as secondary metabolite (Pitt, 1987). It was first discovered in 1965 as a fungal toxin in animals and first isolated from South African *A. ochraceus* isolate (van der Merwe et al., 1965). It occurs in nature in 3 different isoforms as Ochratoxin A, B, and C, among these Ochratoxin A (OTA) is the most potent. OTA is proven to be carcinogen in animals, and has been classified as a class 2B, possible human carcinogen by International Agency for Research (IARC, 1993; Marin-Kuan et al., 2011; Haighton et al., 2012). OTA induces hepatotoxicity, genotoxicity, teratogenicity and cancer. Kidney has been considered as the key target organ of OTA toxicity in most of the mammalian species (Patharajan et al., 2011; Jilani et al., 2012).

Furthermore, investigations showed OTA as a neurotoxic agent (Belmadani et al., 1998a; Sava et al., 2006a). Studies in rodents suggest that OTA crosses the blood brain barrier and gets accumulated within brain and after single administration of OTA dose to mice, highest OTA concentrations were found in the cerebellum followed by the pons and cerebral cortex (Belmadani et al., 1998b; Sava et al., 2006a,b). OTA was also found in ventral mesencephalon, cerebellum, striatum and hippocampus. However, relatively high OTA levels were found hippocampus, a primary site of neurodegeneration in Alzheimer’s disease, with concurrently pronounced OTA neurotoxicity (Belmadani et al., 1998b). Alzheimer’s disease (AD) is the most common form of neurodegenerative disease with factors influencing its development including genetic factors, gender, hypertension, head trauma and chemical exposure which can be characterized by changes the brain that include entorhinal area, neocortex, specific sets of subcortical nuclei and amygdale hippocampus. Studies indicate that p38 mitogen-activated protein kinase (MAPK) could play more than one role in Alzheimer’s Disease and p38 MAPK inhibitors will reduce dementia lead by neurodegeneration (Kim and Choi, 2010; Zlokovic, 2011). Various studies reveal that fundamental biology of Alzheimer’s disease is related to p38 MAPK functions, such as synaptic plasticity, excitotoxicity and tau phosphorylation (Munoz and Ammit, 2010; Wang et al., 2014).

In several reports it was found that OTA activates members of the MAPK family, extracellular signal-regulated kinases 1 and 2. In nanomolar concentrations OTA showed to activate c-jun amino-terminal-kinase (JNK) a second member of MAPK family in MDCK-C7 cells (Doi and Uetsuka, 2011, 2014; Zheng et al., 2013). A drop in expression of these genes is likely to result in decreased antioxidant defense leading to oxidative stress. Yoon et al. (2009) extrapolated the results of oxidative stress in kidney cells, to substantiate that OTA generated ROS in hippocampal HT22 and human neuroblastoma SH-SY5Y cells, varying neuronal cell viability and proteome profiles, but caspase activation and increased phosphorylation of p53 was seen mainly in HT22. In another study conducted by Zhang et al. (2009) showed that OTA induced caspase 9 and 3 activation leading to apoptosis by loss of mitochondrial membrane potential (MMP) in SH-SY5Y cells. In addition, OTA exposure was also found to enhance the expression of hemeoxygenase-1, inducible nitric oxide synthase (iNOS) and modulate the gene profiling analysis of genes involved in calcium homeostasis (Kumar et al., 2012; Sorrenti et al., 2013). *N*-acetylcysteine (NAC) is clinically used to scavenge oxidants directly or indirectly and counteract oxidative stress. Studies show that NAC-promoted survival of sympathetic neurons in PC12 cells independent of glutathione (GSH) elevation requiring ongoing transcription and it is found to prevent apoptosis and promote cell survival through alterations in a variety of intracellular signaling pathways such as c-jun, c-fos and p53 (Yan and Greene, 1998; Crispo et al., 2010; Berniakovich et al., 2012). However, the complex neurobiological mechanism underlying the neurotoxication caused by OTA is not completely modeled *in vitro* and the protective efficacy of NAC on neurotrophic pathways affected by OTA. Hence, the present study was aimed at investigating OTA induced neurotoxicity *in vitro* in Neuro2a cells and its protection with NAC.

## Materials and Methods

### Reagents and Chemicals

Minimum essential medium, penicillin-streptomycin antibiotic solution, 3-(4,5-dimethylthiazol-2-yl)-2,5-diphenyltetrazolium bromide (MTT), 2′,7′ dichlorfluorescein-diacetate, rhodamine 123, sodium dodecyl sulfate (SDS), 2,2-azinobis (3-ethyl-benzothiazoline-6-sulfonic acid; ABTS), glutathione standard, acetylthiocholine iodide, 5,5′-dithiobis(2-nitrobenzoic acid) (DTNB), bovine serum albumin, protease cocktail inhibitor, Ochratoxin A, *N*-acetylcysteine, low melting agarose and Sodium bicarbonate were purchased from Sigma-Aldrich St. Louis, MO, USA. Hydrogen peroxide, sodium chloride, sodium dihydrogen phosphate, disodium hydrogen phosphate were procured from S.D Fine Chemicals, Mumbai, India. RNA isolation kit (RNeasy mini kit) was acquired from Qiagen, Valencia, USA. cDNA synthesis kit (Transcriptor first strand synthesis kit) was bought from Roche, Germany. Horse serum from Life Technologies, USA, and fetal bovine serum was procured from Hyclone, USA.

### Cell Culture and Treatments

Neuro-2a cells supplied by National Centre for Cell Science, Pune, India were used in the present study. The Neuro-2a cells were cultured in minimum essential medium and supplemented with 10% fetal bovine serum (FBS). Streptomycin (100 μg/ml) and penicillin (100 U/ml) was added to the culture media. Cell line was maintained in a humidified atmosphere of 5% CO_2_ and 95% air at 37°C. All the experiments were conducted with 80% confluent cells with more than 95% cell viability and in serum free media. OTA was prepared freshly to add to the cells for incubation of 24 h with or without pretreatment with NAC for 1 h before any experiment as per OECD TG 487 guidelines.

### Cell Viability Assay

Cell viability and plasma membrane damage were assessed by MTT assay and LDH leakage assay as per previous studies (Pandareesh and Anand, 2014).

### Estimation of Intracellular ROS

The cells were cultured in 24 well plates for fluorimetric analysis and treated as mentioned earlier. After treatments, the oxidation-sensitive dye DCFH-DA (0.5 mg/ml) was added to the cells and incubated for 30 min. The cells were then collected after washing twice with PBS and the intracellular ROS formation was detected using Multi-technology plate reader (Plate Chameleon, Type 425-106 s/n 2090137, Finland) at an excitation wavelength of 485 nm and an emission wavelength of 535 nm.

### Estimation of Intracellular Hydroperoxides

Levels of water soluble hydroperoxide in Neuro-2a cells were determined following ferrous iron oxidation with xylenol orange (FOX1) as described previously. After treatments, the cells were collected, washed twice with PBS and lysed in ice-cold PBS and centrifuged at 13,500 × *g* for 10 min at 4°C. Two hundred micrograms of protein in 30 μl was added to 950 μl of FOX1 reagent and incubated at room temperature for 30 min. The reaction mixture was centrifuged at 800 × *g* for 10 min and the optical density of the supernatant was measured at 560 nm.

### Lipid Peroxidation Assay

Malondialdehyde (MDA), a byproduct of lipid peroxidation, was calculated by Ohkawa et al. (1979) method with slight modifications. Neuro-2a cells were cultured in 75 cm^2^ flasks with 1 × 10^6^ cells/ml concentration and incubated at 37°C. When it reached 80% confluence it was treated as described earlier. The cells were collected, washed twice with PBS and lysed in ice-cold 1.15% KCl with 1% Triton X-100 by sonication for 5 min. Aliquots (100 μl) of the cell lysates were mixed with 0.2 ml of 8.1% SDS, 1.5 ml of 20% acetic acid (pH 3.5), 1.5 ml of 0.8% thiobarbituric acid and the volume was brought up to 4.0 ml using distilled water. The contents were boiled for 2 h to develop the color, followed by cooling. The content was centrifuged at 3000 × *g* for 10 min and absorbance of the supernatants was measured at 532 nm. The MDA content was calculated using a molar extinction coefficient of 1.56 × 10^5^ M^-1^ cm^-1^ (Buege and Aust, 1978).

### Measurement of Mitochondrial Membrane Potential (MMP)

OTA induced mitochondrial damage was determined by measuring the MMP using the fluorescent dye rhodamine 123. For fluorimetric analysis cells were seeded in 24 well plates. Rhodamine 123 (10 mg/ml) was added to the cells after the treatment and incubated for 1 h at 37°C. Cells were washed twice with PBS, and collected, fluorescence was detected at an excitation wavelength of 485 nm and an emission wavelength of 535 nm.

### Single Cell Gel Electrophoresis (SCGE) Assay

Nuclear damage induced by OTA via oxidative stress was assessed by alkaline comet assay with minor modifications (Singh et al., 1988). The cells (1 × 10^6^ cells) were seeded in 75 cm^2^ flasks and treated as described previously. Neuro-2a cells were treated with 1 mM concentration of NAC and incubated for 1 h. Later OTA (500 nM) was added and kept for 6 and 24 h. Equal volume of cell suspension (4 × 10^5^) was mixed with 0.5% (w/v) low melting agarose (LMA) in 0.01 M of PBS to the treated cells. The mixture was pipetted on the frosted slides with pre-coating of normal melting agarose 1% (w/v). After the agarose solidified, another 100 μl of 0.5% (w/v) LMA was pipetted on the slides and immersed in lysis buffer (2.5 M NaCl, 100 mM EDTA, 10 mM Tris-HCl buffer, 0.1% SDS and 1% Triton X-100 and 10% DMSO; pH 10.0) for 120 min in dark at 4°C to lyse the cellular and nuclear membranes. The slides were rinsed with unwinding buffer and transferred into an electrophoresis tank containing unwinding buffer (3 M NaOH, 10 mM EDTA; pH 13.0) for denaturing the DNA followed by electrophoresis for 30 min with an electric current of 25 V. The slides were washed twice with neutralizing buffer (0.4 M Tris-HCl; pH 7.5) for 10 min and ethanol treatment was done another 5 min. Ethidium bromide (20 mg/ml) 40 μl was used to stain the slides and DNA damage visualized using fluorescence microscope (Olympus, Japan equipped with Cool SNAP^®^ Pro color digital camera). Appearance of ‘comet’ with fragmented DNA (tail) being separated from undamaged nuclear DNA (head) was seen in damaged cells and measurements were made by Comet Assay IV software to determine the tail movement (%). The results were expressed as percent tail movement.

### DAPI Staining

DAPI assay was performed to reveal the nuclear apoptosis induced by OTA in Neuro-2a cells. The cells were cultured in 35 mm Petri dish. After the treatments, DAPI at a concentration of 100 ng/ml was added to the cells and incubated for 10 min at 37°C, apoptotic nuclei was observed under fluorescence using florescent microscope.

### DNA Damage Protective Activity

DNA damage protective property of NAC against OTA toxicity was checked using pRSET-A plasmid grown in *Escherichia coli*. Plasmid DNA was isolated using QIA prep Spin Mini prep kit. OTA was used to oxidize the plasmid DNA and protection was checked by treating with NAC, it was checked on 1% agarose according to Russo et al. (2000) with minor modifications. The experiment was carried out with 200 ng of pRSET-A plasmid DNA in TE buffer (10 mM Tris-Cl and 1 mM EDTA) pH 8.0. Cell were pretreated with 1 mM NAC for 10 min and then OTA (500 nM) was added to the final volume and incubated for 5 min at room temperature. After the treatment the reaction mixture along with gel loading dye (6X) was loaded on 1% Agarose gel and electrophoresed. Plasmid DNA bands were visualized under the UV-transilluminator and image was captured.

### Total RNA Isolation, c-DNA Synthesis and PCR Analysis

RNeasy spin columns (Qiagen, USA) was used extract total RNA as per manufactures instructions. Total RNA was checked for its purity and integrity by running formaldehyde denaturation gel and quantified by nanodrop. c-DNA was synthesized as per the manufactures instructions (Roche Diagnostics, Germany) and stored at -20°C for further use. Gene expressions of AChE, BDNF, NOS2 and TH were examined by quantitative real-time PCR. GeneTool 1.0 software was used to design primers and synthesized at Imperial Life Sciences (P) Ltd. Haryana, India (**Table 1**). Housekeeping gene used was 18S RNA. for quantitative real-time PCR, PCR reaction used was 2 ng of total RNA converted into c-DNA, 2x Eva green ready mix PCR reagent containing dNTP’s, Taq-Polymerase 10 μl. 10 pmol forward and reverse primers were added to the reaction mixture and final volume made upto 20 μl with PCR grade water. The program cycle was set as follows: thermal profile consist of 10 min of annealing at 50°C, one cycle at 95°C and 5 min of polymerase activation followed by 45 cycles of PCR for 10 s at 95°C and 60°C for 30 sec followed by melt curve analysis performed at 65–95°C with increment of 0.5°C for 10 s to confirm the authenticity of the amplified product by its specific melting temperature (Tm) with the melting curve analysis software of the Bio-Rad CFX-96. The threshold cycle (Ct) of gene of interest and housekeeping gene and the difference between their Ct values (ΔCt) were determined. Relative and normalized gene expression was calculated using Bio-Rad software.

**Table 1:** Primers sequences used for quantitative PCR.

Gene	Primers		Product Size (bp)
18S RNA	Forward -5′AGGCGCGCAAATTACCCACTC 3′	477–497	277
	Reverse-5′GCCCGCTCCCAAGATCCAAC 3′	681–700	
AChE	Forward-5′TCGCAGCCTTTGGGGGAGAC 3′	653–672	227
	Reverse-5′AGCGCCACCTGGGGGACA 3′	862–879	
BDNF	Forward-5′CGGCCCAACGAAGAAAACCATAA 3′	607–629	154
	Reverse-5′GGCGCCGAACCCTCATAGACAT 3′	739–760	
NOS2	Forward -5′GCCCCACGGAGAACAGCAGAG 3′	143–163	284
	Reverse -5′GGGCGGGTCGATGGAGTCAC 3′	407–426	
TH	Forward -5′TCGGAAGCTGATTGCAGAGA 3′	620–639	96
	Reverse -5′TTCCGCTGTGTATTCCACATG 3′	695–715	

### Western Blot Analysis

Total cellular protein was quantified by Lowry et al. (1951) method and 100 μg of protein from each sample was loaded onto 10% gel separated on SDS-PAGE and nitrocellulose membrane was used to transfer proteins using electro blotting apparatus (Cleaver Scientific Ltd, UK) according to previous method (Anand et al., 2012). The membranes were probed with β-actin (ab8229), JNK3 (55A8), phospho-SAPK/JNK (Thr183/Tyr185; 98F2), c-jun (ab32137), phospho c-jun (phospho S63; ab32385) at 1: 1000 dilutions whereas active caspase 3 antibody (ab90437) at 1:400 dilution and incubated at 37°C for 3 h after transfer. TBST was used to wash the membranes four times for 15 min and incubated at room temperature for 2 h in horseradish peroxidase conjugated goat anti-mouse and goat anti-rabbit secondary antibodies (DAKO, Denmark) at a dilution of 1:10,000. The membranes were developed using an enhanced chemiluminescence detection system (ProteoQwest^®^, Sigma) after washing. After developing the membranes they were exposed to x-ray film and band intensity captured and measured using NIH imageJ analysis software.

### Estimation of Antioxidant Status

Neuro-2a cells (1 × 10^6^ cells) were cultured in 75 cm^2^ flasks and treatment given according to previous description. The cells were collected and sonicated in 50 mM ice-cold potassium phosphate buffer containing 2 mM EDTA, pH 7.4 and 0.1% Triton X-100 to lyse them. To remove cell debris the content was centrifuged at 13,000 × *g* for 10 min at 4°C. Protein content was analyzed by Lowry et al. (1951) method. Antioxidant activities of enzymes such as superoxide dismutase (SOD), glutathione peroxidise (GPx) and glutathione reductase (GR) were estimated according to the manufacturer instructions (Randox, Cat no. SD. 125, RS 504, GR 2368, Canada) while catalase (CAT) was estimated by measuring the decay of 6 mM H_2_O_2_ solution at 240 nm by the spectrophotometric degradation method (Aebi, 1984). An extinction coefficient of 43.6 M/cm was used to determine the enzyme activity and values were expressed as mmol H_2_O_2_ degraded/min/mg of protein. DTNB was used to determine GSH which yields a yellow chromophore which was measured spectrophotmetrically at 412 nm. Standard curve prepared with reduced glutathione was used to calculate the concentration of GSH and expressed as μg/mg protein. ABTS cation radical decolorization is the measure of total antioxidant. Decrease in absorbance for 3 min at an interval of 1 min was monitored at 734 nm. Molar extinction coefficient of ABTS^+^ 1.5 × 10^4^ mol^-1^cm^-1^ gives the concentration of total antioxidants (Re et al., 1999).

### Statistical Analysis

The data are expressed as mean standard deviation of the mean (SD). Analysis of date was done by one-way ANOVA followed by Tukey’s *post hoc* test using SPSS version 15 software. Differences at *p* < 0.05 were considered to be significant.

## Results

### OTA Treatment Reduced Neuro-2a Cell Viability

**FIGURE 1:**
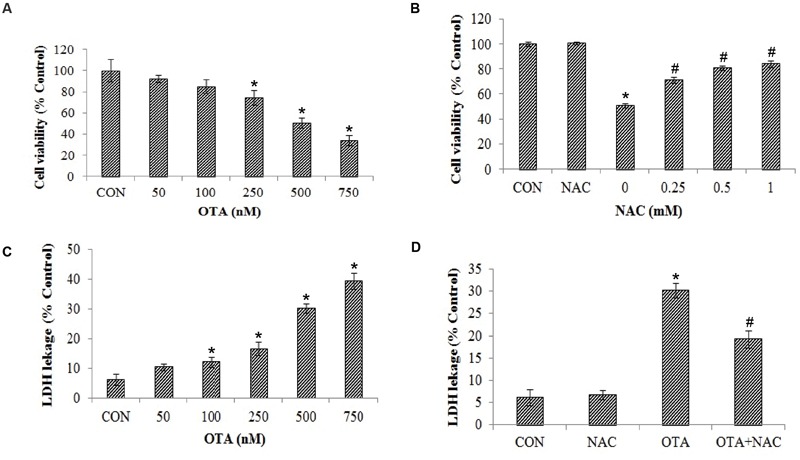
**Cytotoxicity of Ochratoxin A (OTA) in Neuro-2a cells and its reversal by *N*-acetylcysteine (NAC). (A)** Neuro-2a cells were treated without or with various concentrations of OTA (50–750 nM) for 24 h. Cells survival was measured by MTT assay. **(B)** Neuro-2a cells were pretreated without or with NAC (0–1 mM) for 1 h and challenged with different concentrations of OTA for 24 h and viability was accessed by MTT assay. **(C)** Membrane damage was measured by LDH leakage assay. **(D)** Neuro-2a cells were pretreated without or with NAC (1 mM) for 1 h and challenged with OTA (500 nM) for 24 h and viability was accessed by LDH leakage assay. The data represented as mean ± SEM of three independent experiments. ^∗^*p* < 0.05 versus respective control group and ^#^*p* < 0.05 versus respective OTA treated group.

Ochratoxin-A treatment (50–750 nM) reduced the viability of Neuro-2a cells in a dose-dependent manner. The OTA induced toxicity showed 50.75% cell survival rate at 500 nM concentration. The same concentration of OTA was considered as 50% effective dose (ED50) and the same dose was used in subsequent experiments (**Figure 1A**). Further, to determine the toxicity of OTA is mediated via oxidative stress, the cells were pretreated with NAC (0.25–1 mM) for a period of 1 h, followed by treatment with OTA (500 nM) for 24 h. As shown in **Figure 1B**, OTA induced cell death was significantly ameliorated by NAC pretreatment and the viability was restored to the extent of 83.72 % of control cells. These results were further confirmed by LDH leakage assay. The Neuro-2a cells were treated with increasing concentrations of OTA (50–750 nM) for 24 h, which lead to dose-dependent release of LDH into media. A 30.23% LDH release was observed at 500 nM in Neuro-2a cells, respectively, (**Figure 1C**). In contrast, pretreatment with NAC (1 mM) showed a decrease in release of LDH up to 19.26% of total as compared with respective Neuro-2a alone treated group (**Figure 1D**). These results indicate OTA toxicity might be mediated mainly by producing oxidants such as reactive oxygen species, which was effectively reversed by NAC as strong antioxidant molecule.

### OTA Treatment Increased ROS and Hydroperoxide Levels

**FIGURE 2:**
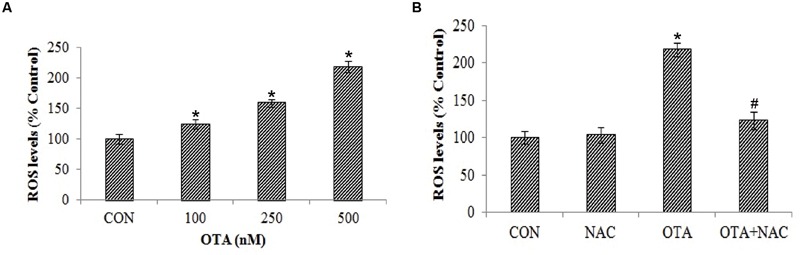
**Effect of OTA on reactive oxygen species (ROS) production in Neuro-2a and its scavenging by NAC. (A)** Neuro-2a cells were treated with various concentrations of OTA (100–500 nM) for 24 h. ROS levels were monitored by using DCFH-DA **(B)**. Neuro-2a cells were pretreated with NAC (1 mM) for 24 h followed by OTA (500 nM) treatment for 24 h and intracellular ROS was quantified by using DCFH-DA. The data represented as mean ± SEM of three independent experiments. ^∗^*p* < 0.05 versus respective control group and ^#^*p* < 0.05 versus respective OTA treated group.

**FIGURE 3:**
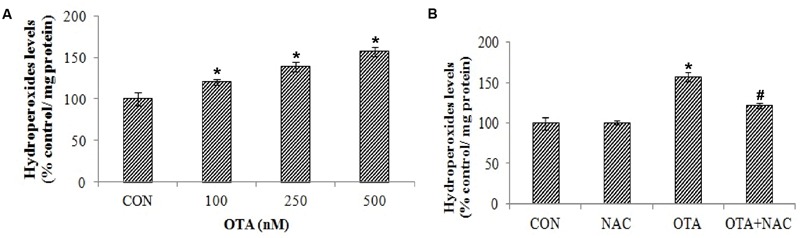
**Effect of OTA on hydroperoxide levels in Neuro-2a and its scavenging by NAC. (A)** Neuro-2a cells were treated with various concentrations of OTA (100–500 nM) for 24 h. Hydroperoxide levels were quantified **(B)**. Neuro-2a cells were pretreated with NAC (1 mM) for 24 h followed by OTA (500 nM) treatment for 24 h and amount of hydroperoxide produced was assessed. The data represented as mean ± SEM of three independent experiments. ^∗^*p* < 0.05 versus respective control group and ^#^*p* < 0.05 versus respective OTA treated group.

Exposure of Neuro-2a cells to OTA elicited ROS and hydrop- eroxide generation in dose dependent manner. Approximately 2 and ∼1.5 fold increased levels of ROS (**Figure 2A**) and hydroperoxide (**Figure 3A**) were observed upon OTA treatment (500 nM) in comparison with their respective control groups. OTA induced ROS and hydroperoxide generation was attenuated significantly (*p* < 0.05) when cells were pretreated with NAC (**Figures 2B** and **3B**). In corroboration with the role of NAC in the reversal of cytotoxicity, generation of ROS and hydroperoxides by OTA treatment was also mitigated by NAC pretreatment (**Figure 2B**).

### OTA Induced ROS Production Induces Lipid Peroxidation, Loss of Mitochondrial Membrane Potential and Nuclear Damage

**FIGURE 4:**
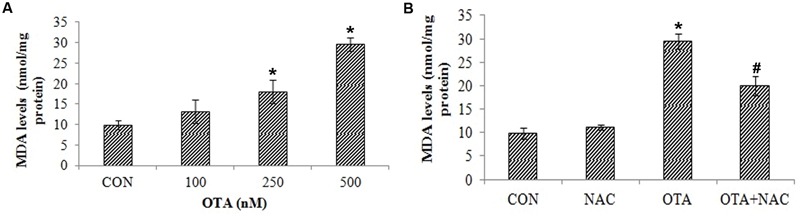
**Effect of OTA treatment on lipid peroxidation and protective role of NAC aginst OTA treatment in Neuro-2a cells. (A)** Neuro-2a cells were treated with various concentrations of OTA (100–500 nM) for 24 h. Hydroperoxide levels were monitored by using thiobarbuturic acid **(B)**. Neuro-2a cells were pretreated with NAC (1 mM) for 24 h followed by OTA (500 nM) treatment for 24 h and amount of MDA product formed was assessed. The data represented as mean ± SEM of three independent experiments. ^∗^*p* < 0.05 versus respective control group and ^#^*p* < 0.05 versus respective OTA treated group.

The effects of OTA on ROS mediated lipid peroxidation, loss of mitochondrial membrane and nuclear damage was determined in Neuro-2a cells. The effects of OTA on lipid peroxidation were measured in terms of MDA levels as a marker of lipid peroxidation. Similar to ROS production, OTA treatment increased the lipid peroxidation in a dose dependent manner (**Figure 4A**). Neuro-2a cells exposed to OTA (500 nM) for 24 h increased the MDA levels significantly by 300.40% relative to the control group. NAC pretreatment had significantly decreased the rate of lipid peroxidation (203.15%) in comparison with OTA treated group (*p* < 0.05; **Figure 4B**).

**FIGURE 5:**
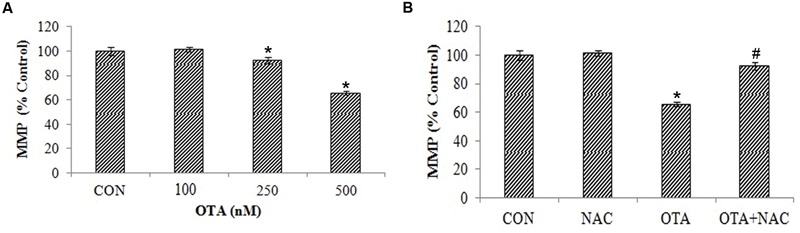
**NAC reversed the mitochondrial membrane potential (MMP) loss induced by OTA treatment in Neuro-2a cells. (A)** Neuro-2a cells were treated without or with various concentrations of OTA (100–500 nM) for 24 h and MMP loss was quantified as fluorescence **(B)**. Neuro-2a cells were pretreated with NAC (1 mM) for 24 h followed by OTA (500 nM) treatment for 24 h and MMP loss was quantified by using rhodamine123. The data represented as mean ± SEM of three independent experiments. ^∗^*p* < 0.05 versus respective control group and ^#^*p* < 0.05 versus respective OTA treated group.

Free radicals attack leads to lipid peroxidation causing a loss of mitochondrial membrane potential in dose dependent manner (**Figure 5A**). This can be analyzed by measuring the accumulation of rhodamine 123, a membrane permeant, cationic fluorescent dye. The rhodamine 123 fluorescence decreased by 65.34% with treatment of OTA compared to control indicating the depolarization of mitochondrial membrane. However, the cells pretreated with NAC prior to OTA exposure, significantly regained florescence intensity up to 92.27% in comparison with its control group (**Figure 5B**).

**FIGURE 6:**
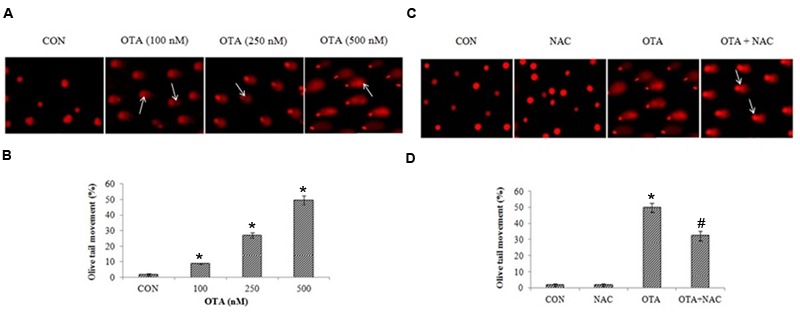
**OTA treatment induced DNA double strand breaks in Neuro-2a cells. (A)** The photomicrographs represent the levels of DNA damage in Neuro-2a cells followed by without or with different concentrations of OTA treatment. **(B)** The tail lengths of the comet was measured in each cell using Comet assay IV software and represented as percent olive tail moment. **(C)** Neuro-2a cells were pretreated with NAC (1 mM) for 24 h followed by OTA (500 nM) treatment for 24 h and DNA damage was monitored and photographed **(D)** Tail lengths of the comet was measured by using Image pro^®^ plus software.

**FIGURE 7:**
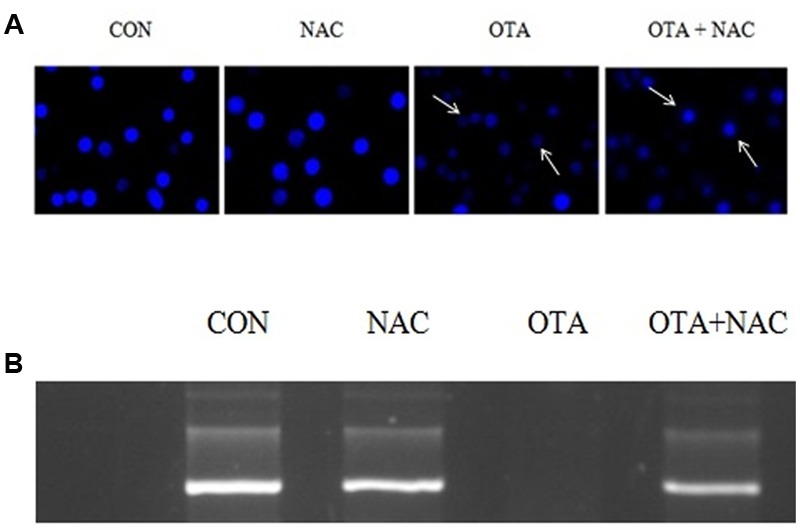
**DNA damage caused by OTA and its protection with NAC. (A)** OTA induced apoptosis in Neuro-2a cells. The photomicrographs represent the levels of apoptotic nuclear damage in Neuro-2a cells pretreated with NAC (1 mM) for 24 h followed by OTA (500 nM) treatment for 24 h and photographed. **(B)** The protective effect of NAC on OTA induced plasmid DNA damage analysis by agarose gel electrophoresis.

Nuclear damage was assessed by SCGE assay. OTA treated (500 nm) cells for 6 h did not show any significant amount of tail length and 24 h incubated cells showed a consistent amount of fragments with a definite tail indicating DNA damage in dose dependent manner (**Figure 6A**). The cells treated with OTA (500 nM) the tail movement was found to be 49.72%. NAC pretreatment has decreased the tail movement by 32.24%. A small tail, little migration of fragments of DNA appeared in NAC treated cells indicating protective efficacy of NAC against OTA induced DNA damage (**Figures 6B,C,D**). The results were confirmed by plasmid DNA damage nick assay. NAC was investigated for its protective efficacy against OTA induced DNA insult using pRSETA plasmid DNA and evaluated on 1% agarose gel, control and NAC treatment (lane 1 and 2) showed two bands on the gel indicating the faster moving band to be native supercoiled circular DNA (ScDNA) and slow moving band to be open circular DNA (OcDNA). Whereas OTA treated plasmid shows clear indication of damage (lane 3) which is reduced considerably on treatment with NAC (lane 4; **Figure 7B**). We further evaluated the effect of OTA on nuclear apoptosis by DAPI staining. The assay is based on the principle that the DAPI stains the nuclei, during the apoptotic event nuclear structures will be disorganized which were observed under fluorescent microscope. DAPI staining showed the presence of apoptotic nuclei with OTA treatment along with or without NAC pretreatment (**Figure 7A**). These results are in agreement with above results of SCGE assay.

### Effect of OTA on Brain Neuronal-Specific Markers Gene-Expression Profiles in Neuro-2a Cells

**FIGURE 8:**
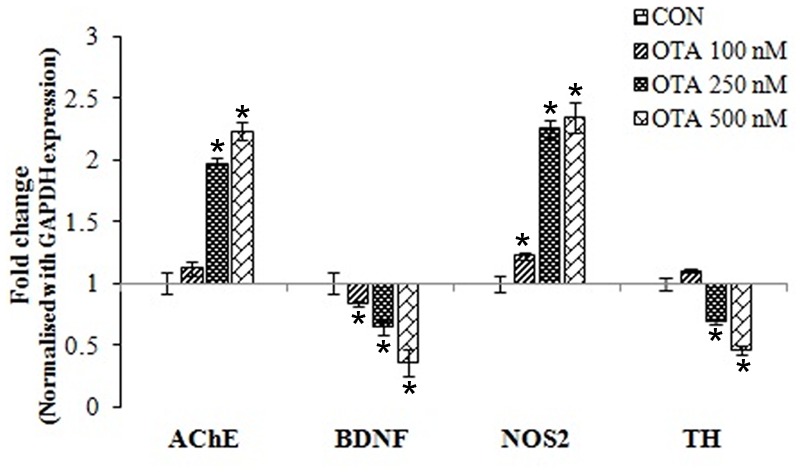
**Gene-expression profiles for neuronal biomarkers (AChE, BDNF, NOS2 and TH) were analyzed in Neuro-2a cells treated without or with various concentrations of OTA (100–500 nM) for 24 h using quantitative real time PCR.** The fold change was calculated based on normalization with GAPDH gene expression. The data represented as mean ± SEM of three independent experiments. ^∗^*p* < 0.05 versus respective control group.

Gene expression profiles of brain neuronal markers (AChE, BDNF, NOS2 and TH) by qRT-PCR in Neuro-2a cells after treatment with OTA in different concentrations for 24 h. Upon OTA treatment, AChE and NOS2 mRNA expression was upregulated whereas BDNF and TH mRNA expression was down regulated in dose dependent manner (**Figure 8**). The gene expression profile results clearly indicate that OTA mediate neurortoxic effect by suppressing specific neuronal markers (like BDNF and TH) and inducing neuronal stress markers (like AChE and NOS2), which play important role in the protection of neuronal cells in the brain.

### OTA Stimulates the Stress-Activated Protein Kinase JNK Cascade

**FIGURE 9:**
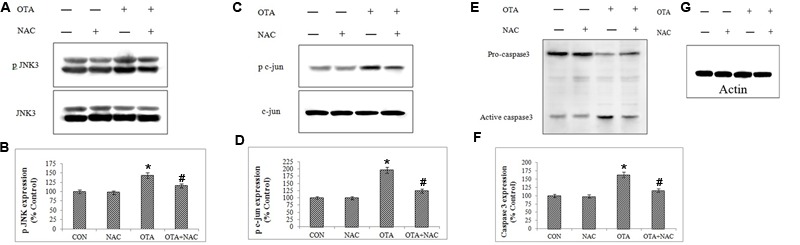
**Protein-expression profiles for apoptotic biomarkers (JNK3, c-jun and caspase3) were analyzed in Neuro-2a cells by immunoblotting (**A,C,E,G**).** The band intensity is calculated by Image-J software **(B,D,F)**. The data represented as mean ± SEM of three independent experiments. ^∗^*p* < 0.05 versus respective control group and ^#^*p* < 0.05 versus respective OTA treated group.

JNK is also known as stress-activated kinase (SAPK) is activated in response to a variety of extracellular insults and proinflammatory cytokines. OTA exposure resulted in the increase in phosphorylation of JNK3. Activation of JNK results in the phosphorylation of c-jun at Ser63 and Ser73. These sites lie within the transactivation domains of c-jun and their phosphorylation serves as an activating event of apoptosis. This was confirmed by cleavage of pro-caspase to active caspase. In order to determine if c-jun was phosphorylated at these sites we performed immunoblots with a phospho-Ser63-specific antibody. The experiments revealed that OTA caused increased c-Jun Ser63 phosphorylation (**Figure 9**) consistent with the increase in JNK3 expression observed in response to OTA treatment. However, NAC pretreatment prevented JNK cascade by decreased phosphorylation of JNK and c-jun.

### Effect of Ochratoxin A on Antioxidant Enzymes Status

SOD, CAT, GPx and GR enzymes, cellular antioxidant levels declined on introduction of OTA in comparison with their respective control cells. However, NAC pretreatment restored antioxidant status in Neuro-2a cells. These results inferred that antioxidant enzyme activity is enhanced by treatment with NAC which is associated with the ROS inhibition. The total antioxidant status and reduced cellular GSH levels was significantly depleted by OTA induced toxicity (*p* < 0.05). The decrease in GSH levels and total antioxidant status was improved by pretreatment with NAC (**Table 2**).

**Table 2:** Effect of NAC on antioxidant status.

	Superoxide dismutase (SOD) (U/mg of protein)	CAT (mM H_2_O_2_ degraded/min/ mg of protein)	GPx (U/mg of protein)	GR (U/mg of protein)	GSH (μM/mg of protein)	TAC (nmol/mg of protein)
CON	1.89 ± 0.09	0.88 ± 0.03	1058.77 ± 57.82	1017.06 ± 51.30	4.05 ± 0.02	42.23 ± 3.12
*N*-acetylcysteine (NAC)	1.91 ± 0.03	0.91 ± 0.05	1050.73 ± 44.37	1046.16 ± 42.64	3.96 ± 0.02	54.87 ± 2.43
Ochratoxin A (OTA)	1.20 ± 0.05^∗^	0.51 ± 0.03^∗^	580.57 ± 36.49^∗^	597.21 ± 29.69^∗^	1.69 ± 0.04^∗^	21.12 ± 3.53^∗^
OTA+NAC	1.68 ± 0.08^#^	0.70 ± 0.05^#^	900.61 ± 33.45^#^	899.32 ± 52.59^#^	2.94 ± 0.05^#^	32.01 ± 2.13^#^

*The data represented as mean ± SEM of three independent experiments. ^∗^*p* < 0.05 versus respective control group and ^#^*p* < 0.05 versus respective OTA treated group.*

## Discussion

Neurotoxic potential of OTA has been implicated via oxidative pathways. Some of the natural antioxidants such as phenolics, provitamins, vitamins, carotenoids, and its derivatives and synthetic compounds like butylated hydroxytoluene (BHT), butylated hydroxyanisole (BHA) could potentially protect the cells against toxicity of various mycotoxins (Galvano et al., 2001). But the molecular mechanisms underlying its neurotoxicity have not yet been abundantly elucidated and data related to protection studies against OTA induced neurotoxicity is limited. Therefore the present study is aimed to further investigate the impact of OTA on biomarkers of oxidative stress, apoptosis and specific neuronal markers which play important role in normal neuronal cell functioning and protective efficacy of NAC against the same. In the present study OTA exhibited neurotoxicity *via* cell mortality, which is confirmed by MTT and LDH leakage assay (**Figure 1**). The mechanism through which OTA induces toxicity *in vitro* may be explained by multiple effects on various sub cellular structures (Bennett and Klich, 2003). The loss of cell viability upon OTA treatment might be due to the loss of cell membrane integrity induced by ROS production leading to LDH leakage. Upon cell membrane damage the intracellular LDH is released to the medium. The cytotoxic activity of OTA on Neuro-2a cells can be correlated with increased extracellular LDH leakage into the medium. These results are in support with earlier reports, which demonstrated that OTA induced oxidative stress leads to cytotoxicity of primary porcine fibroblasts and its effect was ameliorated by antioxidant α-tocopherol (Fusi et al., 2010).

Disruption of the redox balance or oxidative stress due to ROS generation within the cell is not just associated with cell proliferation and signaling but also induces apoptosis (Ramyaa and Padma, 2013; Ramyaa et al., 2014). OTA stimulates DNA and protein nitration demonstrating OTA exposure to be a source of both reactive oxygen and reactive nitrogen species resulting in oxidative stress through various direct and indirect mechanisms (Crowell et al., 2003; Cavin et al., 2007). OTA induces oxidative damage through the generation of hydroxyl radicals via the Fenton reaction as well as *via* flavoprotein NADPH-cytochrome P450 reductase and ferric ion (Fe^3+^) system leading to lipid peroxidation (Hoehler et al., 1996). The by-products found as a result of lipid peroxidation are 4-hydroxy-2-nonenal (HNE), acrolein, crotonaldehyde, aldehydes like malondialdehyde have been widely studied. These malondialdehyde and 4-hydroxynonenol are DNA-damaging agents. Studies have shown that OTA has been reported to cause lipid peroxidation by increasing MDA and formation of HNE-protein adducts (Cavin et al., 2007). In addition, studies have also shown that OTA induces oxidative DNA damage as confirmed by comet assay, plasmid DNA damage assay and micronucleus formation. These findings confirmed that OTA is a potent ROS inducer within mammalian system. (Renzulli et al., 2004; Hibi et al., 2011; Palabiyik et al., 2013).

Results of our study also provided insight into the cytotoxicity of OTA mediated by ROS and hydroperoxides generation in a dose dependent manner (**Figures 2A** and **3A**). The total ROS was measured spectrofluorimetrically using a non-polar, non-ionic fluorescent probe DCFH-DA. The dye DCFH-DA hydrolyses enzymatically by intracellular esterases and crosses cell membranes to produce non-fluorescent DCFH. By the action of ROS, DCFH will be oxidized to highly fluorescent dichlorofluorescein. The resulting fluorescence is an index to measure the ROS levels (LeBel et al., 1992). The fluorescence emitted is directly proportional to the ROS generation induced by OTA. Our results elucidated that toxicity of OTA is due to dose dependent ROS production. Pretreatment with NAC, a strong antioxidant efficiently decreases fluorescence, by neutralizing ROS and hydroperoxides production (**Figures 2B** and **3B**). OTA treatment further led to the formation of lipid peroxides and other free radicals in dose dependent manner (**Figure 4**). The present study shows that ROS generation is one of the underlying mechanisms for OTA induced loss of cell viability and DNA damage.

Mitochondria plays a critical role in explaining the signaling pathways involved in OTA mediated cell death (Vaseva et al., 2012). As mentioned earlier, free radical generation causes lipid peroxidation and increases the permeability of the mitochondrial membrane. This decrease in mitochondrial membrane potential was found to be associated with several apoptosis models and it is estimated to evaluate stress induced apoptotic cell damage (Doi and Uetsuka, 2011). In the present study, OTA treatment led to loss of mitochondrial membrane potential in a dose dependent manner (**Figure 5**), and DNA damage in cells as well as in plasmid DNA (**Figures 6A–D** and **7A,B**). Studies show that p53 dependent cell cycle arrest, DNA repair and apoptosis are associated with oxidative stress (Liu F. et al., 2012; Stachurska et al., 2013). Under all stresses, including oxidative stress, a fraction of cellular p53 traffics to mitochondria and initiates apoptosis (Chaudhari et al., 2009). The major generator of ROS in cells is from mitochondria. Hence, the altered MMP by ROS releases several apoptosis inducing factors (van Gurp et al., 2003) leading to cell death.

Rhodamine 123, a selective mitochondrial, lipophilic and cationic fluorescent dye that partitions into highly negative mitochondrial membrane based on active mitochondria and it can be used to check potential of mitochondrial membrane. The dispersal and accretion of the dye in mitochondria is relative to the degree of mitochondrial membrane potential (Ubl et al., 1996). Leakage of rhodamine 123 into cytosol indicates damage to mitochondrial membrane which then moves out of the plasma membrane and shows decreased intracellular fluorescence. In the current study it was seen that OTA treatment induces dose dependent loss of fluorescence, which is normalized with NAC pretreatment. Our results corroborate with previous reports which state, OTA triggered caspase-9 and caspase-3 activation with mitochondrial membrane potential loss and in human lymphocytes (Assaf et al., 2004).

The neuronal biomarkers AChE, BDNF, NOS2 and TH play pivotal role in mycotoxin mediated toxicity. The gene expression level of these neuronal markers (AChE, BDNF, NOS2 and TH) was monitored by quantitative real time RT-PCR after OTA treatment (**Figure 8**). We found that OTA treatment altered the gene expression profile of neuronal biomarkers confirming the neurotoxic potential of OTA. Earlier studies have shown that alteration in AChE activity is one of the main causes for AD (Talesa, 2001). Lautert et al. (2014) showed that OTA induced acetylcholine esterase (AChE) activity which led to increased cellular oxidative stress levels. A member of the neurotrophin family, brain-derived neurotrophic factor (BDNF) directs neuronal cell survival, promotes growth and differentiation in the developing nervous system. It is also known to be involved in structural and synaptic plasticity (Hu et al., 2011), neuroprotective effects (Saylor and McGinty, 2008), anxiety (Martinowich et al., 2007) and learning and memory (Egan et al., 2003). BDNF expression abnormalities in brain also inspire neuropsychiatric and neurodegenerative diseases (Berton et al., 2006). There are documented studies which have evidence of neurological disease like Alzheimer’s disease, Huntington’s disease, Parkinson’s disease and schizophrenia caused by decrease in expression of BDNF leading to oxidative and apoptotic stress insults (Murer et al., 2001; Caccamo et al., 2010). Hence, the present study, we have examined the effect of OTA on BDNF expression. OTA administration has decreased expression of BDNF in a dose dependent manner. In addition effect of OTA on TH expression was also studied, where the expression of TH was significantly alleviated in a dose dependent manner (**Figure 8**). These results are in support with the earlier reports where the OTA treatment decreased the TH immunoreactivity in fibers of striatum, corpus striatum and substantia nigra by increase in oxidative stress and lipid peroxidation with transient inhibition of oxidative DNA repair activity. This in-turn leads to low levels of striatal dopamine and its metabolites, a biomarker for Parkinson disease and Alzheimer’s disease (Torack and Morris, 1992; Kastner et al., 1993).

One of the major events in neuronal apoptosis is activation of the JNK signaling pathway. JNK, also known as stress-activated protein kinases, represents a group of enzymes activated by exposure of cells to environmental stressors (Whitmarsh and Davis, 1996). JNK is activated through phosphorylating the Thr- and Tyr-residues by upstream MAPK kinases usually MKK4 and/or MKK7 (Ip and Davis, 1998; Dhanasekaran and Reddy, 2008). Activation of JNK phosphorylates the transcription factor c-jun inducing of proapoptotic members of the Bcl-2 family leading to facilitation of the release of cytochrome c (as well as other pro-apoptotic factors) from the intermembrane space of the mitochondria into the cytosol leading to caspases activation, which leads to cellular apoptosis. The present study data clearly demonstrate that OTA induces apoptosis via activation of JNK pathway by increased phosphorylation of JNK followed by c-jun which finally leads to activation of caspase 3 (**Figure 9**). Studies show that expression of p38-MAPK, JNKs, and ERKs has been investigated in mild to severe cases of Alzheimer’s and further causes to the progression of the disease (Liu et al., 2007; Wang et al., 2014). Results of the present study show that NAC has the ability to mitigate the neurotoxic effects of OTA activated via JNK pathway in N-2a cells (**Figure 9**). These results were further supported by chromatin staining with DAPI (**Figure 7A**). Heterogeneous staining pattern appeared after treatment with OTA as compared with control cells. Chromatin condensation or fragmentation is indicated by small bright spots, an alteration typical of apoptosis.

Over production of reactive oxygen species or reduced cellular antioxidant levels leads to oxidative stress. The predominant enzymatic defense systems against OTA induced toxicity are SOD, CAT, GPx and GR are present in all cells and play a crucial role in ameliorating the oxidative damage within the cells. In the present study, Neuro-2a cells exposure to OTA has significantly depleted the levels of these antioxidant enzymes and the toxic effect of OTA was considerably negated by NAC treatment (**Table 2**).

*N*-acetylcysteine targets a diverse array of factors contributing to the pathophysiology of multiple neuropsychiatric disorders including glutamatergic transmission, neurotrophins, apoptosis, mitochondrial function, and inflammatory pathways. Recent studies show that NAC promotes the survival of sympathetic neurons and also helps in transcription by protecting the cells from apoptotic stimuli by depriving trophic factor to enhance neuronal survival (De Vries and De Flora, 1993; Liu J. et al., 2012; Bavarsad Shahripour et al., 2014). It is possible that NAC has protective effects against the OTA induced toxicity by neutralizing excessive ROS production or ROS mediated effects like lipid peroxidation and mitochondrial membrane potential loss, DNA damage and finally apoptosis. NAC has an extensive spectrum of actions and promising applications in neuroprotection in a variety of *in vitro* and *in vivo* models of neuronal death (Karalija et al., 2012; Venkataramana et al., 2014). NAC as a drug, being a xenobiotic directly enters endogenous biochemical processes and crosses the blood brain barrier, has a potential to be treated as cytoprotectent against oxidative stress mediated neuronal damage from OTA and other toxins by its permissible profile of action on multiple operative pathways, and the emergence of positive trial data.

OTA exposure is allied with increase in of oxidative DNA, protein, and lipid damage. Also, it was seen that OTA treatment altered various biological pathways mobilized by oxidative stress. The oxidative stress response resulting from OTA exposure evidenced in the present study is alleviated by antioxidant action of NAC. Finally we conclude that ROS is the upstream signal leading to JNK mediated caspase-dependent apoptosis in OTA induced neurotoxicity in Neuro-2a cells, which was further evidenced by NAC pretreatment.

## Author Contributions

All authors listed, have made substantial, direct and intellectual contribution to the work, and approved it for publication.

## Conflict of Interest Statement

The authors declare that the research was conducted in the absence of any commercial or financial relationships that could be construed as a potential conflict of interest.
